# Association between Visit-to-Visit Glucose Variability and Cognitive Function in Aged Type 2 Diabetic Patients: A Cross-Sectional Study

**DOI:** 10.1371/journal.pone.0132118

**Published:** 2015-07-01

**Authors:** Chulho Kim, Jong-Hee Sohn, Min Uk Jang, Sung-Hun Kim, Moon-Gi Choi, Ohk-Hyun Ryu, Sungwha Lee, Hui-Chul Choi

**Affiliations:** 1 Department of Neurology, Hallym University Sacred Heart Hospital, Chuncheon-si, Republic of Korea; 2 Department of Neurology, College of Medicine, Kangwon National University, Chuncheon, Republic of Korea; 3 Division of Endocrinology and Metabolism, Hallym University Sacred Heart Hospital, Chuncheon-si, Republic of Korea; Cardiff University, UNITED KINGDOM

## Abstract

**Background and Purpose:**

Diabetes is associated with cognitive decline as well as the development of dementia. Although mean blood glucose levels are typically used to assess the status of diabetic patients, glucose variability is also involved in the manifestation of macro- and microvascular complications in this population. Thus, the present study sought to determine whether visit-to-visit glucose variability contributes to cognitive decline in patients with type 2 diabetes.

**Methods:**

The present study assessed 68 patients with type 2 diabetes using several validated neuropsychological measures. All patients had no cerebrovascular disease, history of hypoglycemia, psychiatric conditions, or other medical illnesses. Standard deviations (SDs) and coefficients of variance (CVs) of the patients’ blood glucose (after fasting and 2 hours postprandial; FBS and PP2), and glycated hemoglobin (HbA1c) values were used as indices of glucose variability. The cognitive outcome parameters were transformed with z-scores and entered into a multiple linear regression model that included educational status, age, sex, vascular risk factors, and mean glucose parameters as covariates.

**Results:**

The mean age of the total patient population was 70.9 years; 46 (67.6%) of the patients were men, and the median follow-up duration at our endocrinology outpatient clinic was 4.8 years. The mean FBS and PP2 glucose levels of the patients were 132 mg/dL and 199 mg/dL, respectively, and the mean HbA1c level was 8.0%. A univariable analysis revealed that only the PP2 value was associated with the Mini-Mental State Examination (MMSE) score, and multivariable analysis revealed that a high SD and/or CV for PP2 glucose were associated with low scores on the Rey Complex Figure Copy test and/or the Verbal Learning Test. Additionally, a high SD and a higher CV for HbA1c level were significantly associated with low MMSE and Digit Span test scores even after adjusting for mean HbA1c values.

**Conclusions:**

The present data indicate that a greater degree of visit-to-visit glucose variability influenced specific types of cognitive function in type 2 diabetic patients independently of mean blood glucose levels. Future studies should focus on whether reductions in glycemic variability will improve the cognitive decline observed in type 2 diabetic patients.

## Introduction

Although blood glucose levels affect cognitive function in normal healthy individuals [1,2], those with diabetes are at greater risks of dementia and cognitive decline compared with healthy individuals [3,4]. It has been shown that hyperglycemia [5,6] and hypoglycemic episodes [7] are associated with cognitive changes in diabetic patients, and there is no doubt that optimal glucose control is important for the prevention of cognitive decline in this patient population. However, current guidelines only address the use of average glucose measures, such as glycated hemoglobin (HbA1c) levels [8,9]. However, the Diabetes Control and Complications Trial (DCCT 1993) revealed possible links between glycemic variability and microvascular complications [10]. Furthermore a subsequent study based on DCCT data suggested that long-term rather than short-term fluctuations in glucose levels contribute to the development of retinopathy and nephropathy [11], and several later studies of type 2 diabetic patients demonstrated that indices of glucose variability are associated with macro- and microvascular complications [11–13].

However, the influence of glycemic variability on the risk of diabetes-associated complications remains a matter of debate [12,14]. *In vitro* and *in vivo* studies have shown that high glucose variability is associated with increased production of reactive oxygen species, which expose the vasculature to oxidative stress [15,16], the nervous system is vulnerable to glucose variability [17]. However, it remains unclear whether long-term exposure to glucose variability induces cognitive changes in diabetic patients. A recent systematic review of the relationship between glucose regulation and cognition found that high HbA1c and glucose levels and a greater degree of glucose variability are negatively associated with cognitive function in aged type 2 patients [18]; however, HbA1c accounted for less than 10% of the variance in cognition. Therefore, we aimed to determine whether visit-to-visit (long-term) glucose variability in type 2 diabetic patients affects cognitive performance independently of average glucose parameters.

## Methods

### Subjects

The present study included 68 randomly selected patients who had regularly visited our endocrinology outpatient clinic for at least 2 years and who were 60 years of age or older. All participants had normal daily living activity, had been diagnosed with type 2 diabetes, and had been taking oral hypoglycemic agents since the time of initial diagnosis. Diabetes was defined as having undergone treatment for diabetes or as a non-fasting random serum glucose level ≥200 mg/dL with symptoms corresponding to diabetes. Patients were excluded from the present analyses if they had regularly used insulin treatment, had a history of hypoglycemic episodes that required in-hospital care, had fewer than six measures of glucose parameters evaluated (fasting blood serum [FBS] glucose, 2-hour postprandial blood glucose [PP2], and HbA1c levels) at enrollment, had a medical condition such as congestive heart failure or chronic renal failure that could affect cognition or activities of daily life, and were illiterate, or had a psychiatric illness. Additionally, a Geriatric Depression Scale (GDS) score was obtained for all participants prior to performance of the cognitive evaluations; patients with a GDS score ≥ 11 were excluded from the study. This study was approved by Hallym University Sacred Heart Hospital Institutional Review Board/Ethics Committee (IRB No: 2012–50), and all study participants provided written informed consent.

### Measurements of the glycemic variability indices

The FBS and PP2 glucose assessments were conducted using the glucose hexokinase method (Hitachi Automatic Analyzer 7600, Hitachi Co, Tokyo, Japan), and whole blood HbA1c levels were measured using high-performance liquid chromatography (Bio-Rad VARIANT II TURBO, Hercules, CA, USA). All participants visited our endocrinology outpatient clinic at 3-month intervals, and the FBS glucose, PP2 glucose, and HbA1c values were obtained at the visits.

Visit-to-visit variabilities in these values were calculated retrospectively from electronic medical records (EMRs) of all available serum glucose measures. Standard deviations (SDs) of the values obtained from a self- monitoring blood glucose system or a real-time continuous glucose monitoring system (CGMS) are widely used as indices of glycemic variability indices, but FBS glucose, PP2 glucose, and HbA1c values are the standard measure of diabetic control utilized in real practice. Moreover, visit-to-visit variabilities of these glucose parameters are also used as indicators of between-day variability in diabetic patients [12,19]. The SDs of the FBS glucose, PP2 glucose, and HbA1c values were obtained by routine assessment at each visit, and the SDs and coefficients of variance (CVs) for the FBS and PP2 glucose levels were used as indices of glucose variability in the present study. The CV (%) was calculated by expressing the SDs as percentages of the means: (CV% = SD/mean × 100).

### Evaluation of Cognitive Function

Each of the 68 participants underwent a cognitive function assessment battery conducted by a psychologist who was blinded to the clinical details of the patients. On the morning of the cognitive tests, the patients were instructed to eat breakfast and to take their regular oral hypoglycemic agent; all patients had a tolerable diet, and no specific caloric restrictions were applied. All neurocognitive testing was performed between 10:00 and 11:00 in the morning to minimize short-term variations in blood glucose concentrations and was comprised of the following tests: (1) the validated Korean version of the Mini Mental State Examination (MMSE) to assess general cognitive function, (2) the Verbal Learning Test (VLT) to assess delayed recall and recognition, (3) the Digit Span test to assess concentration and verbal working memory, (4) the Boston Naming Test (BNT) to assess confrontational naming, (5) the Rey Complex Figure Copy Test (RCFT) to assess visual perception and memory, and (6) the Controlled Oral Word Association Test (COWAT) to assess semantic/phonemic fluency. The Korean versions of these cognitive tests have been validated, and the result provided a standard normal distribution chart that was calculated from 447 healthy subjects [20,21]. The z-scores of each cognitive test were calculated as follows: [(each cognitive score—the mean score) / SD], where the mean score and SD were the standardized mean and SD, respectively.

### Confounding Factors

The patient’s demographic variables, the time elapsed since the initial diagnosis of diabetes, and information regarding vascular risk factors were collected from the EMRs of the patients. Hypertension was defined as currently taking antihypertensive medication or an average sitting systolic blood pressure ≥140mmHg or diastolic blood pressure ≥90mmHg. Hyperlipidemia was defined as a fasting total cholesterol level ≥240 mg/dL or current treatment with a lipid-lowering agent. A current smoker was defined as one who smoked one or more cigarettes per day within the previous 6 months; patients who had given up smoking for > 6 months were defined as non-smokers.

### Statistical Analysis

*Pearson*’s correlation test was used to identify the relationship between the z-scores obtained from the individual neurocognitive tests and the glucose variability parameters. *Spearman’s rho* test was used to determine whether the demographic and other categorical variables were correlated with the glycemic indices. Multiple linear regression analysis was used to examine the relationships between the indices of glucose variability and each cognitive score. Independent variables that showed a high variance inflation factor (≥ 8 score) in a regression equation were removed from the final model to minimize multicolinearity. Variables with *P-* values < 0.05 according to univariable analysis and any associated vascular risk factors were entered into the multivariable model. Two-sided *P*-value < 0.05 was considered to indicate statistical significance. A previous study found that the CV of postprandial glucose explained 11.6% of the variance in the MMSE measure [22]. Using these data, the sample size for linear regression was calculated, and it was determined that 62 total participants were needed to reach a statistical power of 80% to detect a significance difference in the CV of postprandial glucose based on the MMSE score, with a two-sided significance level of 5%.

## Results

The characteristics of the 68 patients are presented in Table 1. The mean patient age was 70.9 ± 5.9 years, and 46 (67.6%) of the participants were men, the mean age at diabetes onset was 55.9 ± 9.5 years, and the median follow-up duration at our endocrinology clinic was 4.8 years (interquartile range: 2.4–6.4 years). The median values (interquartile ranges) of the FBS and PP2 glucose measurements and HbA1c assessments per participant were 18 (10–29), 20 (11–29), and 20 (12–28), respectively.

**Table 1:** Baseline characteristics of study subjects.

	Total (n = 68)	
Age, year (mean ±SD)	70.9 (±5.9)	
Female, n (%)	46 (67.6)	
Onset of diabetes, year (median, IQR)	56.0 (49.3–62.0)	
Duration of follow up, year (median, IQR)	4.8 (2.4–6.4)	
Risk factor		
Hypertension, n (%)	47 (69.1)	
Hyperlipidemia, n (%)	30 (44.1)	
Current smoking, n (%)	6 (8.8)	
FBS, number	19 (10–26) *	
Mean ± SD, mg/dL	132 (4)	
PP2, number	20 (11–26) *	
Mean ± SD, mg/dL	199 (±50)	
Hb A1c, number	20 (12–25) *	
Mean ± SD, %	8.0 (±1.2)	
Cognitive test	Median, IQR	z score (95% CI)
MMSE	26 (23–28)	-0.51 (-2.08 - -0.04)
Digit Span**	2 (1–3)	0.03 (-0.67–0.83)
BNT	45 (39–49)	-0.72 (-2.09–1.48)
RCFT	30 (26–34)	-0.69 (-1.71–0.51)
VLT (delayed recall)	4 (2–7)	-0.10 (-0.98 - -1.10)
VLT (recognition)	10 (8–11)	-0.95 (-1.54—-0.08)
COWAT (semantic)	12 (10–15)	-0.84 (-1.34 - -0.09)
COWAT (phonemic)	13 (6–20)	-1.16 (-1.97 - -0.70)

Abbreviations: FBS, fasting blood glucose; PP2, postprandial 2 hour blood glucose; HbA1c, glycated hemoglobin; SD, standard deviation); IQR, interquartile range; z, z-score; MMSE, Mini Mental State Examination (total score,30); BNT, Boston Naming Test (total score, 50); RCFT, Rey Complex Figure Copy Test (total score, 36); VLT, verbal Learning test [total score, delayed recall (12) and recognition (12)]; COWAT, Controlled Oral Word Association Test [total score, semantic (20) and phonemic (15)]

* FBS, PP2 and HbA1c values reported as the median numbers are frequency values, indicating how many glucose measurement had been checked in a participant.

** Digit Span (forward-backward score, total score, 9).

S1 Table summarizes the results of the Pearson’s correlation tests between each cognitive measure and the other parameters. Notably, the duration of follow-up, rather than the duration of diabetes, was associated with the MMSE, Digit Span, and VLT recognition z-scores. According to the univariable analysis, PP2 glucose was the only glucose parameter associated with the MMSE score. Multivariable analysis (Table 2 and S2 Table) revealed that high SDs and high CVs for the PP2 glucose and HbA1c values were associated with low MMSE and Digit Span scores, a high CV for PP2 glucose was associated with a low RCFT score, and a high SD and high CV for PP2 glucose were associated with a low VLT score. The SD and CV for FBS glucose were not associated with any cognitive test scores.

**Table 2:** Linear Regression Analysis results between global cognition, attention, visuospatial and memory scores and glycemic variability parameters.

Variables	Statistical parameters	MMSE	Digit Span	RCFT	VLT (delayed recall)	VLT (recognition)
FBS_SD*	*B*	-0.017	-0.014	-0.024	-0.147	-0.009
	95% CI	-0.042–0.008	-0.029–0.002	-0.060–0.013	-0.393–0.099	-0.025–0.008
FBS_CV*	*B*	-0.028	-0.023	-0.037	-0.205	-0.015
	95% CI	-0.066–0.010	-0.046–0.001	-0.093–0.018	-0.579–0.169	-0.040–0.010
PP2_SD**	*B*	-0.017	-0.007	-0.032	-0.223	-0.005
	95% CI	-0.038–0.004	-0.022–0.007	-0.064–0.001	-0.437–-0.008	-0.019–0.010
PP2_CV**	*B*	-0.036	-0.012	-0.072	-0.482	-0.010
	95% CI	-0.079–0.007	-0.041–0.018	-0.137–-0.007	-0.918–-0.045	-0.040–0.020
HbA1c_SD***	*B*	-1.032	-0.536	-1.118	-1.260	-0.268
	95% CI	-1.773–-0.291	-1.016–-0.056	-2.242–0.006	-8.874–6.354	-0.780–0.245
HbA1c_CV***	*B*	-0.093	-0.046	-0.111	-0.051	-0.030
	95% CI	-0.154–-0.032	-0.087–-0.006	-0.204–-0.017	-0.689–0.587	-0.073–0.012

*B* (regression coefficient), CI (confidence interval). The analysis was adjusted for hypertension, hyperlipidemia, current smoking, and mean glucose parameters. For example, SD or CV of FBS glucose values were adjusted for mean FBS values.

*mean fasting glucose

**mean postprandial 2 hour glucose

***mean glycated hemoglobin

## Discussion

The findings of the present study demonstrated that some degree of cognitive decline was associated with high indices of glucose variability independent of average glucose levels. Furthermore, these associations remained significant after adjusting for previously established risk factors, such as age, years in fulltime education, other demographic factors, and vascular risk factors.

The majority of studies have investigated changes in the cognitive function of diabetic patients from the perspective of long-standing hyperglycemia or hypoglycemic episodes [23,24], while only a few studies have evaluated the association between individual glucose variability and cognitive function in this population. In fact, the impact of glucose variability on cognitive function in diabetic patients has not even been considered in recent prospective studies [25,26]. On the other hand, an in vivo analysis showed that fluctuations in glucose are more damaging to endothelial function compared with a stable, high glucose level [16]. Despite the small sample size and retrospective nature of the present study, these findings support the hypothesis that glucose variability is associated with cognitive changes in type 2 diabetic patients.

Several possible mechanisms may contribute to the cognitive decline associated with glucose fluctuations in diabetic patients. In particular, glycemic variability is associated with an increased production of reactive oxygen species, which in turn, cause glucose-mediated vascular damage in the central nervous system [15,27,28]. An *in vivo* analysis of healthy volunteers and diabetic patients revealed that glucose fluctuations have a more toxic effect on endothelial dysfunction and oxidative stress than does a constant glycemic level [16]. Furthermore, glucose variability influences the occurrence of hypoglycemic episodes, and, conversely, a lower degree of glucose variability is related to fewer hypoglycemic episodes [28–31]. The present data also demonstrated that glucose fluctuations are associated with specific types of cognitive decline. However, it needs to be confirmed whether glucose fluctuations in type 2 diabetic patients affect cognitive function in the mediation of oxidative stress injury or other inflammatory processes.

Patients with a history of hypoglycemic episodes who required in-hospital care were excluded from the present study to eliminate the effects of hypoglycemia on cognitive function. However, it is possible that some patients with subclinical hypoglycemia were included in the final population, and thus the present findings should be interpreted to indicate that patients with high glucose variability exhibited reduced cognition and poor mediation of hypoglycemia.

A variety of glycemic variability indices can be calculated from the within-and between-day analyses of glucose measurements. Within-day glycemic variabilities such as mean amplitude of glycemic excursions using CGMS are associated with cognitive decline in aged type 2 diabetic patients [32,33]. Although the acute fluctuations of within-day glycemic variability provide a comprehensive view of glycemic variance, the CGMS and self-monitoring blood glucose system are more invasive compared with conventional glucose measurements, and it is difficult to perform a longitudinal study using these techniques. On the other hand, visit-to-visit glycemic variability calculated using conventional glucose measures has been effectively used as a measure of between-day variability and was shown to be associated with the risk of future stroke in type 2 diabetic patients [19]. The present study found a possible link between visit-to-visit glycemic variability and cognitive decline in aged type 2 diabetic patients using cognitive tests performed prospectively by a blinded neuropsychologist. However, the blood glucose values were obtained retrospectively from EMRs, and therefore, these associations should be confirmed using longitudinal data.

The present study has several limitations that warrant consideration. First, the presence of coexisting cerebrovascular diseases were not evaluated using magnetic resonance imaging scans, which is important because hippocampal atrophy, silent brain infarcts, and white matter changes are associated with the development of cognitive impairments in diabetic patients [34]. Nevertheless, several comorbid vascular risk factors were investigated thoroughly, and the correlation tests between variability in glucose levels and each of the cognitive outcomes were adjusted for these confounders. Additionally, only patients 60 years or older were included in the present study to minimize the diversity of vascular burdens among the participants. Second, the present study employed a single-center retrospective design, which could have resulted in selection bias, and it relied on retrospective collection of glucose parameters from the EMRs, which may have resulted in recall bias. However, the relatively long follow-up period and the number of glucose parameters used in the present study can be considered strengths that support the observed association between glucose variability and cognitive function. Lastly, hypoglycemic events are obviously related to cognitive decline in diabetic patients [35], and thus patients with a history of hypoglycemic episodes who required in-hospital care were excluded from these analyses. However, asymptomatic hypoglycemic episodes are typically underestimated in observational studies, and this risk increases as glycemic variability increases [36]. Therefore, efforts to differentiate patients with asymptomatic hypoglycemic events should be included in future studies to better define the impact of glycemic variabilities on the risk of cognitive decline.

There were also several strengths to the present study. First, cognitive status can be influenced by fasting; thus, the patients’ dietary habits and use of oral hypoglycemic agents were controlled for prior to the cognitive testing to minimize their effects on cognitive performance. Second, enrollment in the study was restricted to diabetic patients without overt hypoglycemic episodes or a history of taking oral hypoglycemic agents other than insulin to limit the possible effects of hypoglycemic episodes. However, these specific enrollment criteria will eventually lead to selection bias, which would affect the generalizability of these findings to the entire type 2 diabetic patient population.

## Conclusion

The present findings indicate that high glucose variability was associated with cognitive decline independently of mean blood glucose levels. Thus, glucose variability may be a contributor to cognitive decline in type 2 diabetic patients. Further studies should investigate whether reductions in glycemic variability improve cognitive decline in this population.

## Supporting Information

S1 Table Correlation Analysis results between cognitive function test z-scores and other dependent variables. Abbreviation: Correlation, correlation coefficient; N, number of participants. Other abbreviations are presented in Table 1. *Spearman’s rho test
(DOCX)

S2 Table Linear Regression Analysis results between language, executive function scores and glycemic variability parameters. *B* (regression coefficient), CI (confidence interval). The analysis was adjusted for hypertension, hyperlipidemia, current smoking, and mean glucose parameters (*mean fasting glucose, **mean postprandial 2 hour glucose, ***mean glycated hemoglobin). For example, SD or CV of FBS glucose values were adjusted for mean FBS values.
(DOCX)
