# Supplementary material for: Association between Visit-to-Visit Glucose Variability and Cognitive Function in Aged Type 2 Diabetic Patients: A Cross-Sectional Study
Source: PLoS One. 2015 Jul 1;10(7):e0132118. doi: 10.1371/journal.pone.0132118 (PMC4488839; doi:10.1371/journal.pone.0132118)
Supplement: S1 Table — Abbreviation: Correlation, correlation coefficient; N, number of participants. Other abbreviations are presented in Table 1. *Spearman’s rho test (DOCX) [file pone.0132118.s001.docx]

**Supplemental Table 1. Correlation Analysis results between cognitive function test z-scores and other dependent variables.**

|  | Statistical parameters | MMSE | Digit Span | BNT | RCFT | SVLT (delayed recall) | SVLT (recognition) | COWAT (semantic) | COWAT (phonemic) |
| --- | --- | --- | --- | --- | --- | --- | --- | --- | --- |
| Age | Coefficient | 0.110 | 0.176 | -0.159 | 0.250 | -0.093 | 0.103 | 0.055 | -0.130 |
|  | p value | 0.374 | 0.160 | 0.224 | 0.052 | 0.456 | 0.405 | 0.657 | 0.365 |
|  | N | 68 | 65 | 60 | 61 | 67 | 68 | 67 | 51 |
| Education, years | Coefficient | -0.074 | -0.133 | -0.217 | -0.113 | 0.014 | -0.209 | -0.174 | -0.266 |
|  | p value | 0.549 | 0.292 | 0.096 | 0.384 | 0.908 | 0.087 | 0.159 | 0.059 |
|  | N | 68 | 65 | 60 | 61 | 67 | 68 | 67 | 51 |
| Female* | Coefficient | 0.015 | 0.185 | -0.054 | -0.078 | 0.135 | 0.260 | -0.084 | -0.082 |
|  | p value | 0.902 | 0.140 | 0.681 | 0.548 | 0.277 | 0.032 | 0.500 | 0.566 |
|  | N | 68 | 65 | 60 | 61 | 67 | 68 | 67 | 51 |
| Hypertension* | Coefficient | 0.065 | -0.064 | 0.040 | 0.061 | 0.218 | 0.120 | -0.043 | -0.139 |
|  | p value | 0.599 | 0.613 | 0.760 | 0.639 | 0.076 | 0.330 | 0.728 | 0.330 |
|  | N | 68 | 65 | 60 | 61 | 67 | 68 | 67 | 51 |
| Hyperlipidemia* | Coefficient | 0.058 | 0.123 | 0.126 | 0.038 | 0.085 | -0.066 | -0.011 | -0.017 |
|  | p value | 0.638 | 0.331 | 0.338 | 0.769 | 0.494 | 0.591 | 0.930 | 0.903 |
|  | N | 68 | 65 | 60 | 61 | 67 | 68 | 67 | 51 |
| Current smoking* | Coefficient | 0.181 | -0.023 | 0.112 | -0.149 | 0.011 | -0.341 | 0.039 | 0.105 |
|  | p value | 0.140 | 0.855 | 0.395 | 0.251 | 0.931 | 0.004 | 0.753 | 0.461 |
|  | N | 68 | 65 | 60 | 61 | 67 | 68 | 67 | 51 |
| Onset of diabetes | Coefficient | 0.085 | 0.288 | 0.018 | 0.063 | 0.087 | 0.076 | -0.098 | -0.053 |
|  | p value | 0.492 | 0.020 | 0.889 | 0.629 | 0.484 | 0.536 | 0.430 | 0.713 |
|  | N | 68 | 65 | 60 | 61 | 67 | 68 | 67 | 51 |
| Duration of diabetes | Coefficient | -0.019 | -0.200 | -0.131 | 0.122 | -0.166 | -0.015 | 0.151 | -0.037 |
|  | p value | 0.876 | 0.109 | 0.318 | 0.351 | 0.181 | 0.905 | 0.221 | 0.796 |
|  | N | 68 | 65 | 60 | 61 | 67 | 68 | 67 | 51 |
| Follow up duration | Coefficient | 0.243 | 0.288 | -0.083 | 0.153 | -0.118 | 0.281 | 0.206 | -0.075 |
|  | p value | 0.046 | 0.020 | 0.527 | 0.239 | 0.341 | 0.020 | 0.095 | 0.601 |
|  | N | 68 | 65 | 60 | 61 | 67 | 68 | 67 | 51 |
| FBS mean | Coefficient | -0.127 | 0.048 | -0.053 | -0.096 | 0.035 | -0.028 | -0.006 | -0.046 |
|  | p value | 0.303 | 0.707 | 0.686 | 0.462 | 0.781 | 0.822 | 0.961 | 0.750 |
|  | N | 68 | 65 | 60 | 61 | 67 | 68 | 67 | 51 |
| FBS SD | Coefficient | -0.198 | -0.110 | 0.071 | -0.196 | -0.068 | -0.154 | -0.076 | -0.120 |
|  | p value | 0.105 | 0.384 | 0.589 | 0.130 | 0.582 | 0.209 | 0.540 | 0.403 |
|  | N | 68 | 65 | 60 | 61 | 67 | 68 | 67 | 51 |
| FBS CV | Coefficient | -0.203 | -0.170 | 0.120 | -0.213 | -0.079 | -0.201 | -0.093 | -0.118 |
|  | p value | 0.098 | 0.176 | 0.360 | 0.099 | 0.524 | 0.100 | 0.453 | 0.410 |
|  | N | 68 | 65 | 60 | 61 | 67 | 68 | 67 | 51 |
| PP2 mean | Coefficient | -0.355 | -0.082 | -0.071 | -0.185 | 0.091 | -0.103 | -0.003 | -0.018 |
|  | p value | 0.003 | 0.514 | 0.588 | 0.154 | 0.463 | 0.402 | 0.984 | 0.903 |
|  | N | 68 | 65 | 60 | 61 | 67 | 68 | 67 | 51 |
| PP2 SD | Coefficient | -0.356 | -0.149 | -0.075 | -0.321 | -0.149 | -0.131 | -0.045 | -0.024 |
|  | p value | 0.003 | 0.238 | 0.568 | 0.012 | 0.229 | 0.287 | 0.718 | 0.867 |
|  | N | 68 | 65 | 60 | 61 | 67 | 68 | 67 | 51 |
| PP2 CV | Coefficient | -0.198 | -0.100 | -0.044 | -0.302 | -0.250 | -0.114 | -0.044 | -0.035 |
|  | p value | 0.105 | 0.429 | 0.736 | 0.018 | 0.042 | 0.354 | 0.724 | 0.808 |
|  | N | 68 | 65 | 60 | 61 | 67 | 68 | 67 | 51 |
| HbA1c mean | Coefficient | -0.074 | 0.047 | -0.187 | 0.012 | -0.082 | -0.021 | 0.086 | 0.001 |
|  | p value | 0.549 | 0.707 | 0.152 | 0.928 | 0.510 | 0.867 | 0.490 | 0.996 |
|  | N | 68 | 65 | 60 | 61 | 67 | 68 | 67 | 51 |
| HbA1c SD | Coefficient | -0.313 | -0.220 | 0.032 | -0.228 | -0.065 | -0.153 | 0.074 | 0.132 |
|  | p value | 0.009 | 0.079 | 0.807 | 0.077 | 0.604 | 0.213 | 0.550 | 0.357 |
|  | N | 68 | 65 | 60 | 61 | 67 | 68 | 67 | 51 |
| HbA1c CV | Coefficient | -0.348 | -0.261 | 0.077 | -0.296 | -0.027 | -0.208 | 0.042 | 0.120 |
|  | p value | 0.004 | 0.036 | 0.561 | 0.020 | 0.827 | 0.088 | 0.737 | 0.403 |
|  | N | 68 | 65 | 60 | 61 | 67 | 68 | 67 | 51 |

Abbreviation: Correlation, correlation coefficient; N, number of participants. Other abbreviations are presented in Table 1.


**Spearman’*s rho test
