# Supplementary material for: Association between Visit-to-Visit Glucose Variability and Cognitive Function in Aged Type 2 Diabetic Patients: A Cross-Sectional Study
Source: PLoS One. 2015 Jul 1;10(7):e0132118. doi: 10.1371/journal.pone.0132118 (PMC4488839; doi:10.1371/journal.pone.0132118)
Supplement: S2 Table — B (regression coefficient), CI (confidence interval). The analysis was adjusted for hypertension, hyperlipidemia, current smoking, and mean glucose parameters (*mean fasting glucose, **mean postprandial 2 hour glucose, ***mean glycated hemoglobin). For example, SD or CV of FBS glucose values were adjusted for mean FBS values. (DOCX) [file pone.0132118.s002.docx]

**Supplemental Table 2. Linear Regression Analysis results between language, executive function scores and glycemic variability parameters.**

| Variables | Statistical parameters | BNT | COWAT (semantic) | COWAT (phonemic) |
| --- | --- | --- | --- | --- |
| FBS_SD* | *B* | -0.023 | -0.008 | -0.009 |
|  | 95% CI | -0.014–0.059 | -0.025–0.010 | -0.025–0.007 |
| FBS_CV* | *B* | 0.038 | -0.012 | -0.013 |
|  | 95% CI | -0.018–0.094 | -0.039–0.014 | -0.037–0.012 |
| PP2_SD** | *B* | -0.006 | -0.003 | -0.002 |
|  | 95% CI | -0.038–0.026 | -0.019–0.013 | -0.018–0.015 |
| PP2_CV** | *B* | -0.012 | -0.006 | -0.006 |
|  | 95% CI | -0.078–0.054 | -0.038–0.027 | -0.039–-0.027 |
| HbA1c_SD*** | *B* | 0.857 | 0.053 | 0.217 |
|  | 95% CI | -0.239–1.954 | -0.488–0.594 | -0.294–0.728 |
| HbA1c_CV*** | *B* | 0.074 | 0.001 | 0.014 |
|  | 95% CI | -0.021–-0.169 | -0.044–-0.046 | -0.030–-0.057 |

*B* (regression coefficient), CI (confidence interval).

The analysis was adjusted for hypertension, hyperlipidemia, current smoking, and mean glucose parameters (*mean fasting glucose, **mean postprandial 2 hour glucose, ***mean glycated hemoglobin). For example, SD or CV of FBS glucose values were adjusted for mean FBS values.
